# Uridine and its role in metabolic diseases, tumors, and neurodegenerative diseases

**DOI:** 10.3389/fphys.2024.1360891

**Published:** 2024-02-29

**Authors:** Yueyuan Yang, Yahong Ye, Yingfeng Deng, Ling Gao

**Affiliations:** ^1^ Department of Endocrinology, Renmin Hospital of Wuhan University, Wuhan, China; ^2^ Department of Internal Medicine, QuanZhou Women’s and Children’s Hospital, QuanZhou, China; ^3^ Department of Diabetes and Cancer Metabolism, City of Hope, Duarte, CA, United States

**Keywords:** uridine, metabolic diseases, circadian rhythm, diabetes, O-GlcNAc, obesity, neurodegenerative diseases

## Abstract

Uridine is a pyrimidine nucleoside found in plasma and cerebrospinal fluid with a concentration higher than the other nucleosides. As a simple metabolite, uridine plays a pivotal role in various biological processes. In addition to nucleic acid synthesis, uridine is critical to glycogen synthesis through the formation of uridine diphosphate glucose in which promotes the production of UDP-GlcNAc in the hexosamine biosynthetic pathway and supplies UDP-GlcNAc for O-GlcNAcylation. This process can regulate protein modification and affect its function. Moreover, Uridine has an effect on body temperature and circadian rhythms, which can regulate the metabolic rate and the expression of metabolic genes. Abnormal levels of blood uridine have been found in people with diabetes and obesity, suggesting a link of uridine dysregulation and metabolic disorders. At present, the role of uridine in glucose metabolism and lipid metabolism is controversial, and the mechanism is not clear, but it shows the trend of long-term damage and short-term benefit. Therefore, maintaining uridine homeostasis is essential for maintaining basic functions and normal metabolism. This article summarizes the latest findings about the metabolic effects of uridine and the potential of uridine metabolism as therapeutic target in treatment of metabolic disorders.

## 1 Introduction

Uridine is a pyrimidine nucleoside found in plasma and cerebrospinal fluid with a concentration higher than the other nucleosides (Dobolyi et al., 2011; Altaweraqi et al., 2020). As a simple metabolite, uridine plays a pivotal role in various biological processes, including macromolecule synthesis, circadian rhythms, inflammatory response (Jeengar et al., 2017), antioxidant process (Lai et al., 2023), and aging (Jiang and Zhao, 2022; Zhang et al., 2022). Plasma uridine enters cells through nucleoside transporter. In addition to nucleic acid synthesis (Yamamoto et al., 2011), uridine is critical to glycogen synthesis through the formation of uridine diphosphate glucose (UDPG) (Roach et al., 2012). Uridine also promotes the production of UDP-GlcNAc in the hexosamine biosynthetic pathway (HBP), which supplies UDP-GlcNAc for O-GlcNAcylation (referred to as O-GlcNAc), a posttranslational modification on the hydroxyl groups of serine/threonine residues catalyzed by O-GlcNAc transferase (OGT) (Banerjee et al., 2016; D Alessandris et al., 2004). Uridine promotes the formation of cell membranes and synaptic structures in neurons, rejuvenates aged stem cells, stimulates regeneration of various tissues, and even have anti-aging effects (Liu et al., 2022). It is shown that uridine has an effect on body temperature (Peters et al., 1987a; Peters et al., 1987b) and circadian rhythms (Zhang et al., 2018). Uridine can also reduce oxidative stress and inflammation by inhibiting the MAPK and NF-kB signaling pathways under pathological conditions (Luo et al., 2021). Therefore, maintaining moderate levels of uridine, especially plasma uridine levels, is critical for keeping cellular basic functions.

**FIGURE 1:**
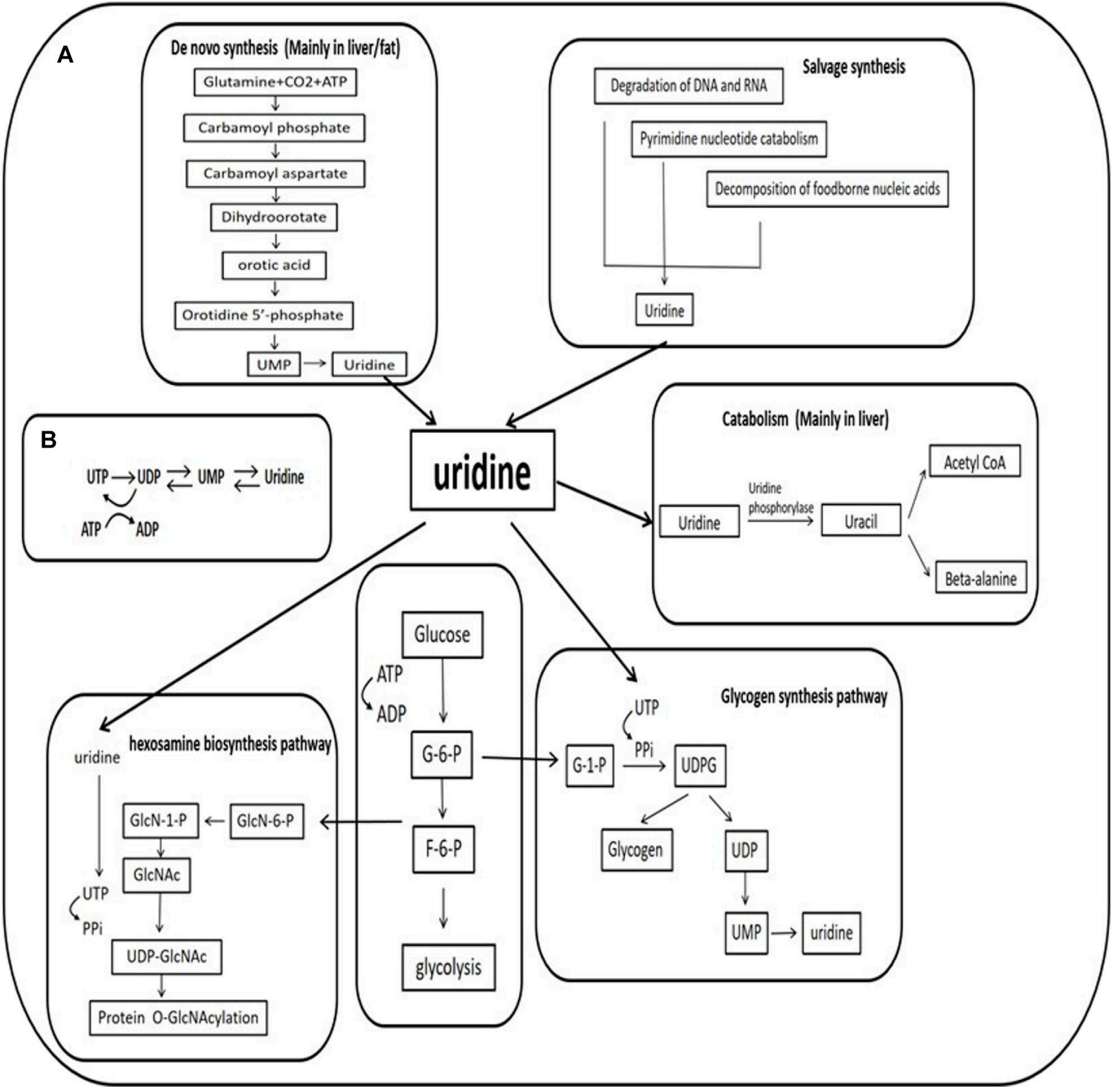
Uridine and its metabolism **(A)**. The mechanism of uridine synthesis and catabolism and the role of uridine in the hexosamine biosynthetic pathway and glycogen synthesis **(B)**. Relationship between ATP consumption and uridine. UTP is produced by the phosphorylation of UDP with ATP used as phosphate donor, a decrease in ATP concentration results in decreased phosphorylation of UDP to UTP, leading to increased UDP and uridine-5′-monophosphate (UMP). These changes accelerate the degradation of uracil nucleotides (UTP→UDP→UMP→ uridine). G-6-P: glucose-6-phosphate; F-6-P: fructose-6-phosphate; GlcN:glucosamine; GlcNAc:N-acetyl glucosamine; UDP:uridine-5′-diphosphate; UTP:uridine-5′-triphosphate; PPi:inorganic pyrophosphate; ADP:cytidine -5′-diphosphate; ATP:cytidine -5′-triphosphate.

The circulating plasma concentration of uridine is strictly controlled between 3–8 μM in different species and individuals (Yamamoto et al., 2011). Under physiological conditions, uridine is mainly produced in the liver (during normal feeding) and adipose tissue (during fasting) through *de novo* synthesis. The clearance of blood uridine is mediated through bile (Deng et al., 2017). The balance between production and clearance determines the homeostatic level of circulating uridine (Ohno et al., 2008; Le et al., 2013) (Figure 1). Uridine uptake is via the nucleoside transport system (Tetsuya Yamamoto Yuji et al., 2000; Altaweraqi et al., 2020). Intracellular uridine levels are associated with ATP consumption and glycogen synthesis (Yamamoto et al., 2011; Chen et al., 2014). Changes in plasma uridine levels are associated with neurodegenerative diseases (de Leeuw et al., 2020), diabetes (Yamamoto et al., 2010; Dudzinska, 2011), and obesity (Kohli et al., 2018). Studies in rodents report that chronic high plasma uridine leads to fatty liver and impaired glucose tolerance (Urasaki et al., 2016), suggesting a causal role of uridine in the progression of those metabolic disorders. This review will focus on the metabolic effects of uridine to reveal the pathophysiological relevance of uridine to metabolic disorders.

## 2 Uridine in the regulation of body temperature and circadian rhythm

### 2.1 Uridine and body temperature

**FIGURE 2:**
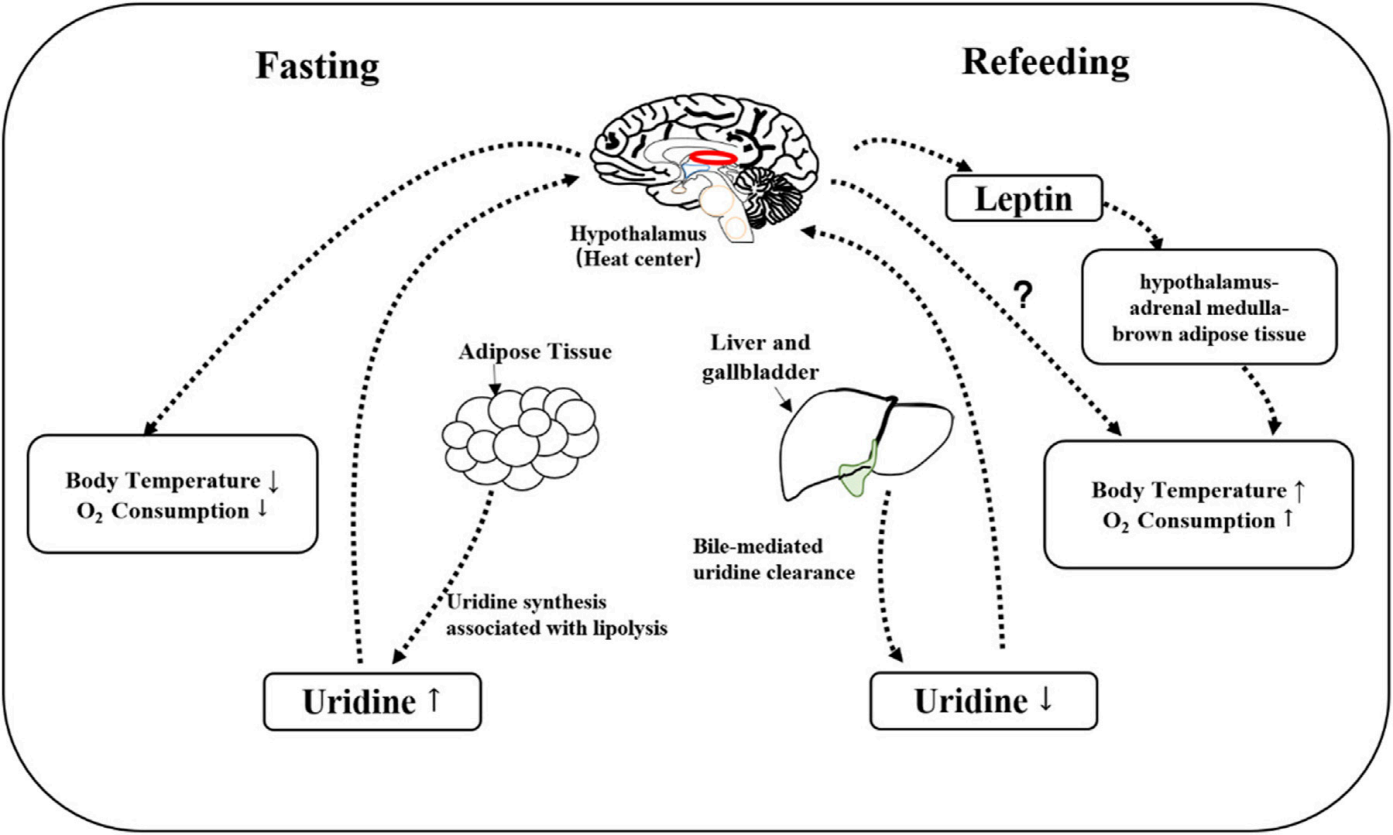
Mechanism of uridine involved in thermoregulation. During fasting, uridine is synthesized mainly by adipose tissue, a process that accompanies the breakdown of fat. The elevated plasma uridine is expected to signal to hypothalamus so that the oxygen consumption and body temperature will decrease. After refeeding, plasma uridine level is decreased through bile, which allows the recovery of oxygen consumption and body temperature. Leptin is not necessary for uridine-induced hypothermia, but leptin-deficiency slows down the recovery of body temperature post uridine administration.

The physiological fluctuations in blood uridine levels can result in changes in body temperature. Elevated plasma uridine levels in fasted mice are accompanied by a decrease in body temperature, which is restored through uridine clearance mediated by bile excretion after refeeding (Deng et al., 2017). Uridine, as a simple metabolite that can cross the blood-brain barrier, is also crucial for maintaining brain function through sustained supply (Dobolyi et al., 2011). The specific molecular mechanism linking peripheral plasma uridine and hypothalamus-mediated temperature regulation is unclear. Compared to wild-type mice fed a high-fat diet (HFD), ob/ob mice (deficient in functional leptin) show a significant delay in body temperature recovery after uridine-induced hypothermia (Deng et al., 2017), suggesting leptin might be involved in body temperature control by uridine. This is consistent with the finding that leptin acts through the hypothalamus-adrenal medulla-brown adipose tissue axis (Fischer et al., 2020; Perry et al., 2020). However, leptin deficiency does not prevent uridine-induced hypothermia (Figure 2).

Uridine-induced change in body temperature is dose- and species-dependent. A low dose of uridine (100 mg/kg) causes a mild increase in body temperature in rodents (Peters et al., 1987a), while high doses (500–3,500 mg/kg) cause severe hypothermia. In contrast, uridine (300–700 mg/kg) causes fever in humans and rabbits (Peters et al., 1987b). It is suggested that uridine-mediated thermoregulation is through its degradation products. Uridine phosphorylase 1 (UPase1), a key enzyme in uridine degradation, is expressed in multiple organs and tissues (Wang et al., 2020; Gonçalves da Silva et al., 2021; Lai et al., 2023). Inhibition of Upase1 prevents the temperature change by uridine in rabbits and rodents (Le et al., 2013). Consistently, the administration of uridine degradative products, such as dihydrouracil and aminoacetyl-β-alanine induces changes in body temperature (Peters et al., 1987b). However, it remains unclear whether the differential response to uridine in rodents and rabbits is simply a species differences or a dose-dependent effect. So far, no studies reported a use of uridine at the dose of or over 1,000 mg/kg in humans or rabbits. Body temperature is regulated by heat production and heat dissipation in mammals. Uridine-mediated fall of body temperature in mice is attributed to reduced metabolic rate and heat production which results from reduced energy demand and oxygen consumption (Deng et al., 2017). Conversely, uridine-induced hyperthermia in rabbits and humans is associated with increased heat production, which results from an upward shift of the set point for body temperature at hypothalamus (Agnati et al., 1986).

It is of great clinical significance to study the impact of uridine on body temperature. It has been demonstrated in mice that the regulation of body temperature by uridine is related to metabolic rate, in which leptin is also involved. Therefore, uridine might be used to treat obesity through its impact on basal metabolic rate. Meanwhile, high-dose oral uridine can reverse 5-fluorouracil (5-FU)-induced leukopenia within several weeks, which subsequently reverses 5-FU-induced bone marrow suppression. This action of uridine enables the use of high-dose 5-FU for cancer treatment (van Groeningen et al., 1989; Ma et al., 2017). However, high doses of uridine can cause fever in humans, an effect that contradicts the use of uridine to combat the side effects of 5-FU. Further studies are warranted to understand the mechanism of uridine-induced fever before uridine can be used as a treatment to alleviate the side effects of anticancer drugs such as 5-FU.

### 2.2 Uridine and circadian rhythm

Food and light are the timing cues for the circadian clocks that are reset in a daily cycle (Xin et al., 2021). Since plasma uridine exhibits diurnal fluctuation (el Kouni et al., 1990) and uridine modifies feeding behavior (Hanssen et al., 2023), plasma uridine might play a critical role in circadian rhythm. The circulating uridine is elevated during fasting, which promotes the synthesis of uridine diphosphate (UDP) in the central nervous system (Cansev, 2006; Ipata, 2011). UDP stimulates the orexigenic agouti-related protein/neuropeptide Y (AgRP/NPY) neurons in the hypothalamic arcuate nucleus, which increases appetite and promotes food intake (Steculorum et al., 2015). It has been shown that the level of plasma uridine was positively correlated with the degree of hunger and food intake (Hanssen et al., 2023), and decreases in proportion with food intake. Obesity can alter this regulation. Thus, circulating uridine is involved in energy homeostasis and the development of obesity (as discussed in section 5.2 below).

Plasma uridine levels in light cycle are higher than dark cycle in mice, which is concordant with increased activity of Upase1 in the liver at night, the key enzyme for uridine degradation (el Kouni et al., 1990). Uridine supplementation changes the fluctuation pattern of plasma uridine and causes altered rhythmic expression of many genes that are involved in lipid, glucose, and nucleotide metabolism (Zhang et al., 2018; Liu et al., 2019) (Table1). When uridine is supplemented at night, the expression of genes for bile acid transport and cholesterol excretion are increased compared to uridine supplemented during the day. In contrast, the expression of genes for cholesterol absorption is decreased by uridine supplemented at night compared to daytime. It appears possible to use uridine supplements at night to treat hypercholesterolemia in mice (Zhang et al., 2018; Liu et al., 2019). The circadian rhythm of rodents is not consistent with that of humans, but it still has guiding significance for the application of uridine in humans. Another study suggests that daytime uridine supplementation inhibits lipid synthesis, reduces polyunsaturated fatty acid synthesis and increases the proportion of saturated fatty acids by inhibiting the expression of Acyl-CoA synthetase long-chain family member 4 (ACSL4) (Lai et al., 2023), an enzyme is known associated with obesity and fatty liver (Zhang et al., 2018). Thus, it is critical to choose right time for uridine supplementation (such as when uridine phosphorylase activity is low) to achieve better therapeutic outcomes.

**TABLE 1:** Genes whose expression is altered after day/night uridine supplementation.

Gene name	Physiological role
SLC29A1	Uridine transport
UPP,UGT1A1	Uridine degradation
DHODH, UMPS	*De novo* synthesis of uridine
FASN	NEFA synthesis
LCAT	Cholesterol transport
G6PC,PC,PCK1	Glucose metabolism
GSK3B	Glycogen synthesis
GLUT2	Glucose transport
FXR,SHP	Bile acid metabolism in liver
ASBT, IBABP, NPC1L1, ABCG8	Bile acid metabolism in ileum
SLC29A1, DHODH, UMPS, UPP, RRM 2, CMPK 2	Nucleotide metabolism in duodenum

## 3 The effect of uridine on protein modification and its related diseases

**FIGURE 3:**
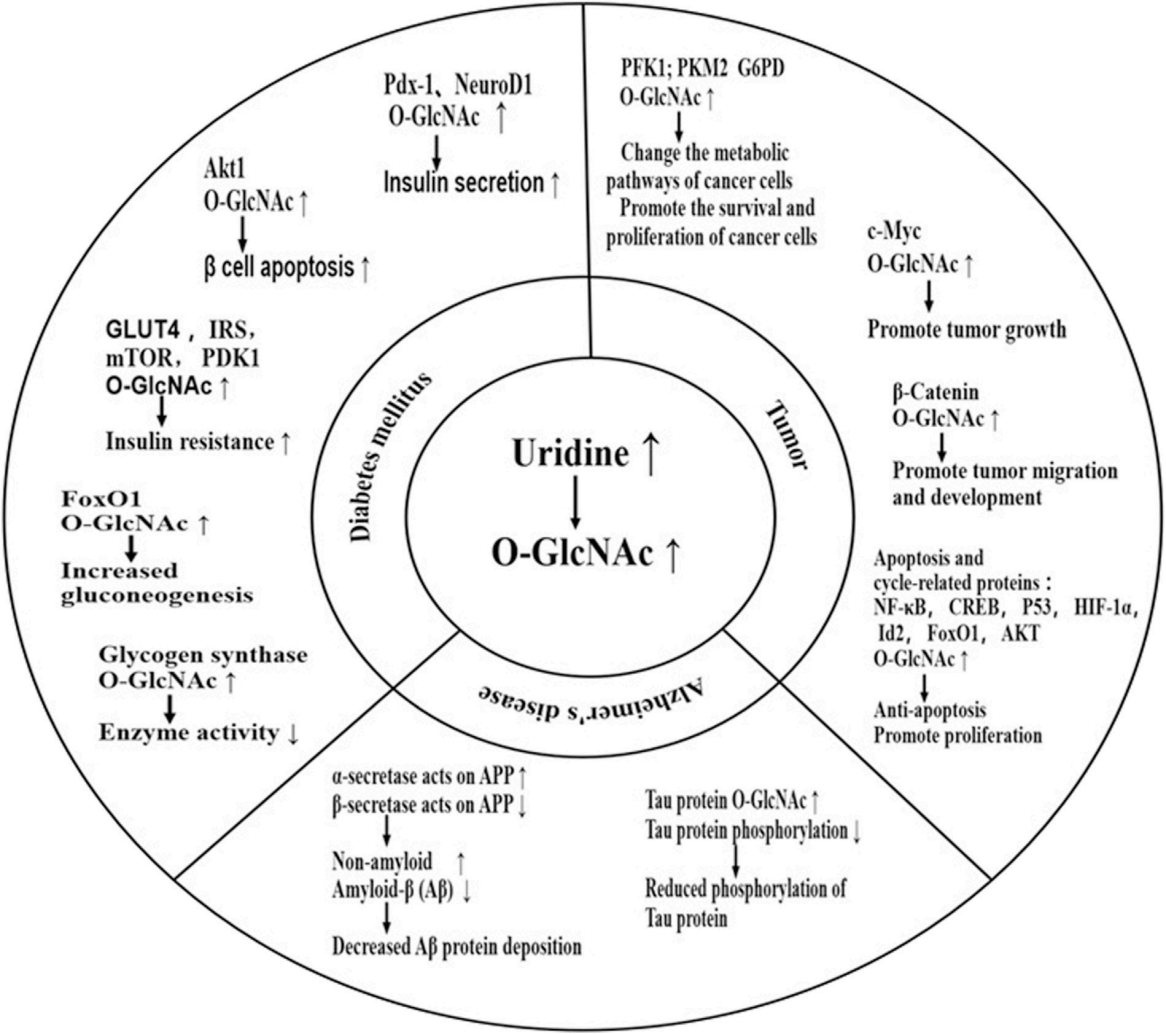
Effect of uridine on disease through O-GlcNAcylation. Uridine can impact diseases by increasing O-GlcNAc of important functional proteins. Current research focuses mainly on diabetes, Alzheimer’s disease, and cancer. Elevated O-GlcNAc levels of specific proteins can worsen blood glucose status by promoting βcell apoptosis, exacerbating insulin resistance, inhibiting glycogen synthesis, promoting gluconeogenesis, among other mechanisms. However, in the short term, it can also promote insulin secretion, partially explaining the differences in the effects of urinary nucleosides on diabetic patients in the short and long term. The typical pathological manifestation of Alzheimer’s disease is hyperphosphorylation of Tau protein and deposition of β-amyloid protein. Supplementation with uridine can provide neuroprotection by increasing O-GlcNAc of Tau protein, reducing Tau phosphorylation, and preventing Tau aggregation. It can also shift the processing of amyloid precursor protein (APP) towards the non-amyloidogenic pathway mediated by α-secretase and away from the amyloidogenic pathway mediated by β-secretase, reducing the production and deposition of β-amyloid protein-induced neurotoxicity. However, high levels of O-GlcNAc can promote tumor development by promoting tumor cell proliferation, inhibiting tumor cell apoptosis, and promoting tumor cell migration. PDX-1:pancreatic and duodenal homeobox-1; NeuroD1:neurogenic differentiation 1; AKT: kinase B; G6PD: Glucose-6-phosphate dehydrogenase; PDK1: phosphoinositide-dependent kinase 1; PFK1: Phosphofructokinase-1; PKM2: pyruvate kinase M2 isoform; IRS: insulin receptor substrate; APP: Amyloid precursor protein; FoxO1:forkhead box O1; HIF-1α:hypoxia-inducible factor 1 α; CREB:cAMP response element-binding protein; Id2:inhibitor of differentiation 2.

Uridine is known to affect protein metabolism through O-GlcNAc modification (Zachara et al., 2015). Supplementation of uridine increases cellular level of UDP-GlcNAc, which leads to an increase in protein O-GlcNAcylation. This modification alters the physical property and biological activity of proteins, leading to a shift in proteins’ function, a potential mechanism for various diseases. Moreover, many O-GlcNAcylation sites are located at or near the phosphorylation sites in the same protein, indicating a competition of O-GlcNAcylation and phosphorylation, which could affect the propagation of phosphorylation events of signaling proteins (Wells et al., 2001). O-GlcNAc modification has been found in almost all functional proteins, including those involved in transcription, translation, and structural composition (Zachara et al., 2015). Current research on O-GlcNAcylation is focused on its significance to neurodegenerative diseases, diabetes (emphasized in Section 6), and tumor (Peterson and Hart, 2016; Su et al., 2020) (Figure 3).

### 3.1 Uridine and tumor

Uridine metabolism plays a critical role in tumorogenesis by supplying UDP-GlcNAc (Cao et al., 2016). Elevated protein O-GlcNAcylation promotes tumorigenesis, whereas reduced protein O-GlcNAcylation lowers tumor incidence and increases cancer cell death (Zachara et al., 2015; Wang et al., 2020). The increase in protein O-GlcNAcylaiton has been observed in various cancer cells, including prostate, breast, lung, colon, and liver cancer cells. Oncogenic signals activate O-GlcNAc transferase (OGT) via the Akt/mTOR pathway. Whereas O-GlcNAcylation of various transcription factors leads to increased cell proliferation, decreased cell death, and activation of cell invasion. O-GlcNAcylation enhances the inhibition of e-cadherin by the transcription factor Snail, which makes cells more prone to dissemination (Zhu et al., 2012). Similarly, O-GlcNAcylation of β-Catenin, the key regulator of intracellular adhesion, promotes tumor migration and development. Conversely, O-GlcNAcylaiton of NF-κB and c-Myc reduces their inhibitory activity, leading to reduced protein turnover and increased cell survival and proliferation (Lam et al., 2021). In addition to regulating cell migration through modifying signaling pathways, O-GlcNAcylation also promotes cancer progression through its impact on cancer cell metabolism. It is reported that O-GlcNAcylation of phosphofructokinase 1 (PFK1) decreases its activity, resulting in a shift of metabolic flux to the pentose phosphate pathway, which increases glutathione and enhance the resistance of cancer cells to oxidative stress (Wurtman, 2017).

In addition, under conditions of glucose restriction, cancer cells can upregulate the expression of UPP1 through the Kras-MAPK pathway, promoting the breakdown and utilization of uridine as a source of nutrients and energy (Nwosu et al., 2023). Because plasma uridine level is positively associated tissue protein O-GlcNAcylation content and UPP1 expression, strategies that aim to alter plasma/tissue uridine concentration and disrupt the utilization of uridine by cancer might be potential treatments for cancer (Banerjee et al., 2016).

Overall, uridine promotes cancer development. This is achieved by using uridine as an energy supplier and changing the key protein O-GlcNAcylation to improve the proliferation, spread and survival of cancer cells.

### 3.2 Uridine and AD

Exogenous uridine supplementation has been shown beneficial for patients with Alzheimer’s disease (AD) (de Leeuw et al., 2020). Animal studies also found that oral administration of uridine prodrug PN401 improves novel object recognition impairment in AD mice (Saydoff et al., 2013). It is suggested that the increase of protein O-GlcNAcylation by uridine is part of the mechanism for the beneficial effect of uridine observed for AD.

Phosphorylation of tau protein and amyloid precursor protein (APP) mediated β-amyloid (Aβ) plaque deposition directly contribute to the pathogenesis of AD (Connolly et al., 1996). The O-GlcNAcylation levels of Tau protein are negatively correlated with its phosphorylation. Levels of total protein O-GlcNAcylation are reduced in the brain tissue of AD patients. Upregulation of Tau protein O-GlcNAcylation levels not only prevents its toxic hyperphosphorylation but also stabilizes its structure, thereby reducing the formation of toxic PHF-Tau (Banerjee et al., 2016). The increase in APP protein O-GlcNAcylation activates the α-secretase process, leading to an increase in soluble amyloid precursor protein α (sAPPα), which inhibits the secretion and accumulation of Aβin brain. Together these data suggest that the increase in protein O-GlcNAcylation may be beneficial for patients with neurodegenerative diseases (Nie et al., 2019). In addition to Tau and APP proteins, O-GlcNAcylation has been observed on proteins involved in processes including signal transduction, transcription, and proteasome degradation.

Uridine supplementation not only increases protein O-GlcNAcylation, but also promotes synaptic membrane synthesis in the cerebral cortex and the hippocampus. Uridine is the substrate for synthesis of cytidine triphosphate, the nucleotide used for production of CDP-choline and CDP-ethanolamine. The increase of CDP-Choline and CDP-ethanolamine production thereby conceivably increases the number of synapses in AD and improves synaptic function (Wurtman et al., 2009; Wurtman, 2017). In addition to AD, uridine has been shown neuroprotective for Parkinson’s and Huntington’s disease in animal models. Treatment with PN401 reduces the loss of dopamine neurons induced by 1-methyl-4-phenyl-1,2,3,6-tetrahydropyridine (MPTP), an inhibitor of mitochondrial complex I, in Parkinson’s disease mouse models (Richardson et al., 2007). PN401 also prevents motor impairment, neurodegeneration and death induced by complex II inhibitors in two Huntington’s disease mouse models (Davidoff, 2006). A single drug can benefit neurodegenerative diseases in multiple ways, making uridine a great potential as a therapeutic agent.

## 4 Uridine and peripheral neuropathy

Uridine exerts profound impact on the peripheral nervous system. Multiple lines of evidence suggest that uridine promotes the growth of nerve cells. Uridine treatment increases neurite outgrowth and branching in neuronal pheochromocytoma (PC12)cells (Pooler et al., 2005). Animal studies also demonstrated a dose-dependent decrease by uridine of apoptotic markers Caspase-3, oxidative markers myeloperoxidase (MPO) and malondialdehyde (MDA) in sciatic nerve tissue of rats with sciatic nerve injury. Mechanistically, uridine serves as a precursor for the synthesis of cytidine-5′-diphosphocholine (CDP-choline), a rate-limiting endogenous intermediate in phospholipid synthesis, which is necessary for nerve growth (Khezri et al., 2021).

Diabetic peripheral neuropathy is a type of neuropathy frequently observed in metabolic diseases. Previous studies have found that 6 months of oral uridine treatment improves nerve function in patients with diabetic neuropathy, which is reflected by increased nerve conduction velocity, nerve fiber regeneration, and myelin sheath surface area and axonal thickness (Gallai et al., 1992). The nutritive and reparative effects of uridine on neurons is through uridine-mediated synthesis of membrane components such as CDP-choline, phosphatidylinositol, and phosphatidylcholine (Dempsey and Raghavendra Rao, 2003). Meanwhile, uridine also exerts its beneficial effects on diabetic neuropathy by promoting glycogen synthesis via the formation of uridine diphosphate glucose, which reduces local glucose accumulation and mitigates the toxic effects of sorbitol on neuronal cells (Akamine et al., 2018).

## 5 Uridine and obesity

### 5.1 Uridine and fatty liver

The relationship between uridine and fatty liver is not set in stone. Uridine supplementation under different conditions leads to opposite conclusions. Short-term uridine supplementation reverses drug-induced (e.g., tamoxifen (Le et al., 2014), Zalcitabine (Lebrecht et al., 2007)), hepatic steatosis (Le et al., 2013). It is suggested that the synthesis of membrane phospholipids is stimulated by uridine and those drug, which reduces the accumulation of triglyceride in cells, thereby decreases the incidence of fatty liver. Meanwhile, overexpression of Upase1 in hepatocytes leads to a decrease in blood uridine levels but leads to fatty liver (Le et al., 2013). In contrast to mouse models with altered uridine metabolism through genetic modification of Upase1, wild type mice after short-term uridine supplementation have increased rates of fatty acid beta-oxidation and are resistant to hepatic steatosis. However,long-term (16 weeks) uridine supplementation induces fatty liver due to the inhibition of liver-specific fatty acid binding protein 1 (FABP1). This may be related to the upregulation of liver P2Y6 receptors leading to the downregulation of PPARα (Jain et al., 2020; Wu et al., 2022). Deficiency of FABP1 is known to promote excessive accumulation of fatty acids in the liver (Guzman et al., 2013; Martin et al., 2015; Urasaki et al., 2016).

### 5.2 Uridine is associated with obesity by regulating energy intake, lipid storage and breakdown

Obesity is a metabolic disorder characterized by excessive expansion of fat mass. An increasing body of research suggests that uridine may be linked to obesity through its involvement in energy intake, storage and expenditure, and may be a potential target for treating obesity. Uridine levels were higher in the fasting state than after a meal. High levels of plasma uridine are suggested to generate the sensation of hunger and promote feeding behavior by stimulating the feeding center (P2Y6-Dependent AgRP Neurons in hypothalamic). With energy intake, plasma uridine and uridine-stimulated sense of hunger decreased in proportion until the end of feeding behavior (Hanssen et al., 2023). Fasting uridine in obese patients is higher compared with healthy people (Steculorum et al., 2015), and the dynamic decline of postprandial uridine is weakened (Kohli et al., 2018). This indicates that obesity disrupts the plasma uridine homeostasis and may be associated with the development of obesity by promoting energy intake. Whether obesity can be reversed by regulating uridine is a topic worthy of further study.

Uridine can also regulate the lipid content in adipose tissue. Long-term supplementation of uridine to mice on a regular diet leads to an increase in body weight (Urasaki et al., 2016). Recent research indicates that blood cells, the brain, lungs, immune cells, and other tissues all have the potential to utilize uridine as a source of nutrition and energy through glycolysis. This unregulated capacity, coupled with chronic energy accumulation, promotes lipid synthesis and contributes to the explanation for obesity, fatty liver, and diabetes resulting from long-term uridine supplementation (Skinner et al., 2023). On the other hand, uridine supplementation reduces weight gain in mice fed on high-fat diet (HFD) (Liu et al., 2019). This difference may be related to the fat content in the diet and the obesity status, with leptin possibly playing a role. The knockout of UDP-activated P2Y6 receptors in adipose tissue exerts resistance to diet-induced obesity through the JNK-PPARα-PGC1α axis (Jain et al., 2020). Meanwhile, the overexpression of mitochondrial complex MIC19 in the liver enhances mitochondrial cristae formation, mitochondrial respiration, and fatty acid oxidation, while suppressing gluconeogenesis, resulting in a similar resistance to diet-induced obesity and an improvement in glucose homeostasis (Sohn et al., 2023). It is suggested that uridine-mediated prevention of weight gain is associated with reprogramming of genes for uridine and lipid metabolism.

Adipose tissue is the major source of uridine supply in fasted state (Deng et al., 2017). The time of uridine synthesis in adipose tissue is in accordance with lipolysis, suggesting a link between uridine synthesis and lipolysis. X-box binding protein 1 (Xbp1) is a transcription factor activated in response to endoplasmic reticulum (ER) stress. Adipocyte selective Xbp1 overexpression stimulates uridine synthesis through the enzyme CAD (a multifunctional protein composed of glutamine-dependent amidotransferase, aspartate carbamoyltransferase, and dihydroorotase). The increased activity of uridine synthesis by Xbp1 is suggested to promote the breakdown of TG in adipose tissue (Deng et al., 2018). The relationship between uridine and obesity is a comprehensive reflection of energy intake, lipid storage and decomposition. In summary, within a short period, both exogenous uridine supplementation and uridine synthesis in adipose tissue appear to protect mice from obesity. Chronic supplementation of uridine may potentially promote the occurrence of obesity. Whether the differential effect of uridine on lipid metabolism is related to the caloric intake status and body fat status of the subjects still needs further research in order to correctly understand the causal relationship and drug value of uridine and obesity.

## 6 The effect of uridine on glycemic control

**FIGURE 4:**
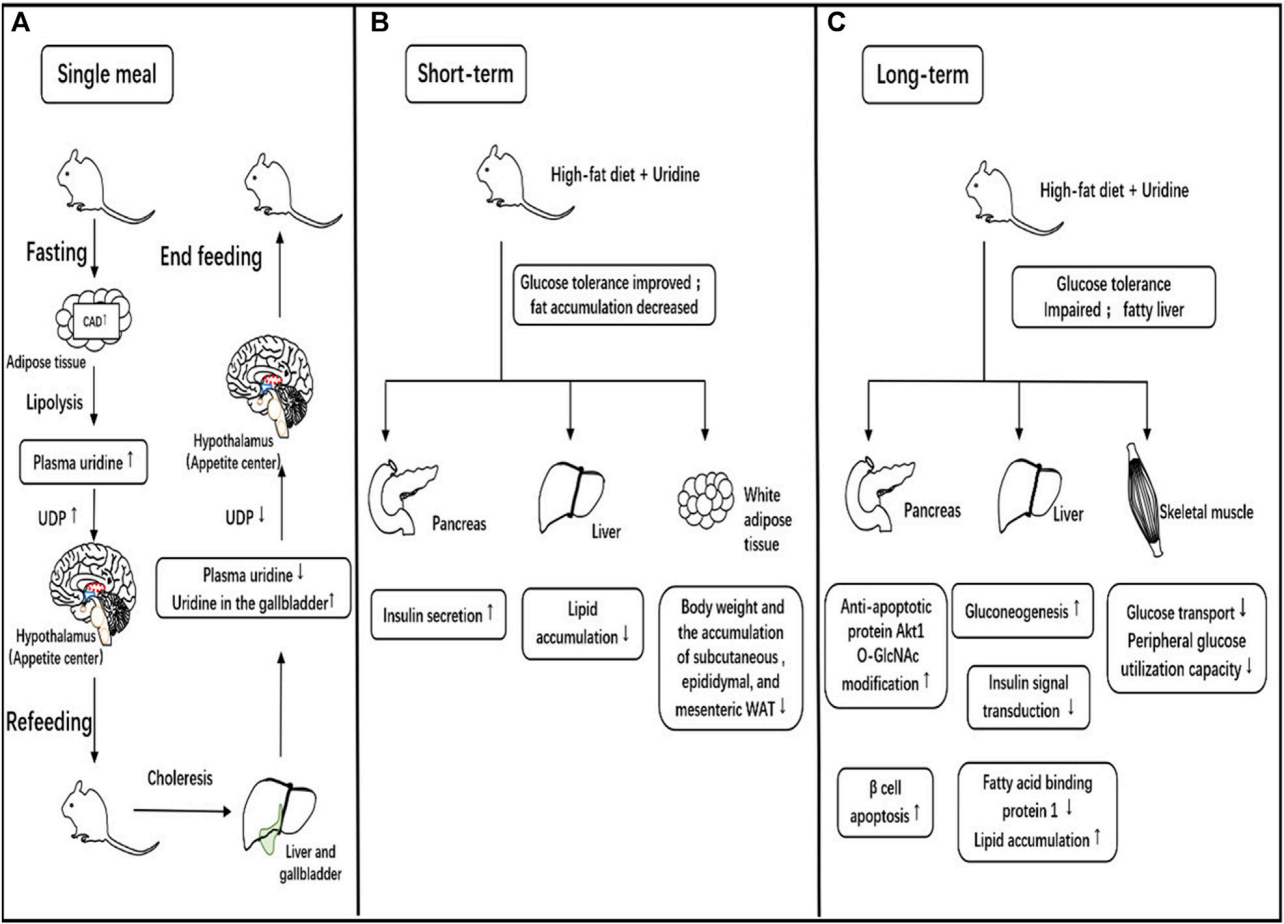
Relationship between uridine and eating behavior, glucose, and lipid metabolism. **(A)**. The role of uridine in a single meal. During fasting, the expression of CAD, a key enzyme in the synthesis of uridine consisting of glutamine-dependent carbamoyl phosphate synthase, aspartate carbamyltransferase and dihydrogen rotamase, in adipose tissue increases, leading to increased uridine synthesis and release into the blood. Uridine diphosphate (UDP) in the central nervous system is synthesized directly dependent on peripheral circulating uridine levels, and increased UDP synthesis stimulates the appetite center to produce hunger and promote eating. Eating promotes bile clearance, which lowers blood uridine levels while increasing uridine concentration in the gallbladder. The decrease in blood uridine concentration reduces UDP synthesis in the central nervous system, leading to a decrease in hunger and cessation of eating. **(B)**. Short-term effects of uridine supplementation on glucose/lipid metabolism. In animal studies, short-term uridine supplementation in high-fat-fed mice promotes insulin secretion and improves glucose tolerance. It can reduce fat accumulation in the liver and alleviate drug-induced fatty liver. It also results in decreased white adipose tissue in multiple locations and weight loss. **(C)**. Long-term effects of uridine supplementation on glucose/lipid metabolism. In animal studies, long-term uridine supplementation in high-fat-fed mice promotes pancreatic beta-cell apoptosis, increases hepatic gluconeogenesis, reduces effective insulin signaling and decreases peripheral utilization of glucose leading to impaired glucose tolerance. Meanwhile, long-term uridine supplementation in mice results in liver fat accumulation and the development of fatty liver.

Uridine is closely related to glucose homeostasis. Changes in gut microbiota are positively correlated with the elevation of uridine in its metabolic products and the presence of impaired fasting glucose, although the mechanism is not yet clear (Nogal et al., 2023). Plasma uridine concentrations are increased inpatients with type 1 and type 2 diabetes (Belosludtseva et al., 2022). However, similar to lipid metabolism, the relationship between uridine and glycemic control has a similar dual side (Figure 4). Uridine promotes glycogen synthesis, and short-term (within 4 weeks) uridine supplementation improves glucose tolerance in mice (Deng et al., 2018; Belosludtseva et al., 2022). In contrast, long-term uridine supplementation increases blood glucose levels and triggers insulin resistance in mice (Urasaki et al., 2014).

### 6.1 The effect of uridine on β cell function mediated by O-GlcNAcylation

Pancreatic β-cells are responsible for secreting insulin, hypoglycemic hormone. The functionality of β-cells determines the category and prognosis of diabetes. Gene expression of insulin is regulated by transcription factors Pdx-1 (pancreatic and duodenal homeobox-1), NeuroD1 (neurogenic differentiation 1), and MafA (V-maf musculoaponeurotic fifibrosarcoma oncogene homolog A). O-GlcNAcylation modification has been shown to increase insulin gene expression and secretion by enhancing the affinity of PDX-1 for DNA (Konrad and Kudlow, 2002; Banerjee et al., 2016). In addition, extracellular nucleotides, such as UTP, UDP, and UDP-glucose conjugates, activate P2 receptors on pancreatic β-cells. Upon P2Y6 receptor is stimulated, the accumulation of IP_3_ increases the cytoplasmic-free Ca^2+^, which activates protein kinase C (PKC) and enhances insulin secretion (Parandeh et al., 2008).

However, long-term elevation of total protein O-GlcNAcylation within β-cells is detrimental. Studies have shown that under high-glucose conditions, the increase in O-GlcNAcylation of certain proteins is associated with β-cell death. Glucosamine treatment induces mouse β-cell apoptosis by increasing the Ser473 O-GlcNAcylation of the anti-apoptotic protein Akt1, a modification concomitnatly decreases Akt1 phosphorylation (Kang et al., 2008). This may be one of the mechanisms by which long-term uridine supplementation leads to impaired glucose tolerance.

### 6.2 The effect of uridine on insulin resistance mediated by O-GlcNAc modification

Insulin resistance is the main cause of type 2 diabetes (T2D). Blood uridine levels in T2D patients are higher than non-diabetic individuals, and blood uridine levels are positively correlated with insulin resistance (HOMA-R) (Yamamoto et al., 2010), suggesting uridine as a potential biomarker for insulin resistance. Proper functioning of insulin signal in cell is necessary for the glucose-lowering effect of insulin. Urasaki.et al. found that uridine supplementation increases O-GlcNAcylation levels of insulin receptor substrates (IRS), Akt, mammalian target of rapamycin (mTOR) and p70S6 kinase (p70S6K), which are key components of insulin signaling propagation and modification in liver (Le et al., 2014). O-GlcNAcylation of those components diminishes the cellular response to insulin, resulting in insulin resistance (D Alessandris et al., 2004; Issad et al., 2010). Meanwhile, increased O-GlcNAcylation of glucose transporter 4 (GLUT4) and/or GLUT4-associated proteins reduces glucose uptake by skeletal muscle cells, which precipitates insulin resistance (Hawkins et al., 1997; Boström et al., 2012; Kang et al., 2012). Reducing the formation of uridine or promoting the catabolism to lower the level of uridine may be one of the ways to improve insulin resistance.

### 6.3 Uridine and vascular complications of diabetes mellitus

Uridine adenosine tetraphosphate (Up4A), a synthetic product of uridine, is an endothelium-derived vasoconstrictive factor (EDCF). It can stimulate the proliferation and migration of vascular smooth muscle cell (VSMC) through the extracellular signal-regulated kinases 1 and 2 (ERK1/2) pathway, which plays a dominant role in the formation of atherosclerotic lesions (Wiedon et al., 2012). The high uridine status of diabetes mellitus further promotes this pathological process. Moreover uridine-induced O-GlcNAcylation has been detected for many regulators of vascular homeostasis such as protein kinase C (PKC), phosphatidylinositol 3-kinase (PI3K), and endothelial nitric oxide synthase (eNOS) (De Vriese et al., 2000). The increase in eNOS O-GlcNAcylation impairs its function and reduces nitric oxide (NO) release. NO relaxes vascular smooth muscle, dilates blood vessels, and increases blood flow. The reduction of NO will decrease local blood flow, causing relative hypoxia, oxidative stress, and vascular damage (Aulak et al., 2020). Overall, diabetic patients have higher uridine levels than healthy individuals, which is expected to exacerbate diabetic vascular complications, by promoting the formation of pathological cells and exacerbating local hypoxia. Whether lowering plasma uridine levels will prevent or delay the onset of diabetic vascular diseases warrants further study.

## 7 Summary

Uridine is a versatile metabolite that plays a role in various metabolic processes. Although the significance of uridine to metabolic disease remains elusive, multiple studies have indicated disrupted uridine homeostasis is involved in the onset and development of diabetes, neurodegeneration, fatty liver, and obesity. Since uridine administration study in rodents reveals a complex impact of uridine on glucose and lipid metabolism, it is instrumental to understand the underlying mechanisms.

In summary, uridine has been linked to the metabolism of proteins, carbohydrates and lipids. Study focusing on the role of uridine in metabolic regulation will likely provide new insights for the diagnosis and treatment of metabolic diseases.
